# KIF5A and ALS: a clinical and genetic description of a case series and review of literature

**DOI:** 10.1007/s10072-026-08885-w

**Published:** 2026-02-28

**Authors:** Anna D’Amico, Roberta Cucunato, Giuseppe Schirò, Giuseppe Salemi, Paolo Ragonese, Vincenzo La Bella, Paolo Aridon, Marco D’Amelio

**Affiliations:** 1 Department of Biomedicine, Neuroscience, and Advanced Diagnostics, University of Palermo, Palermo, Italy; 2 Neurology and Multiple Sclerosis Center, Unità Operativa Complessa (UOC), Foundation Institute “G. Giglio”, Cefalù, 90015 Italy

**Keywords:** ALS, MND, KIF5A, Mutations, Variants, Genes

## Abstract

Approximately 10% of ALS (amyotrophic lateral sclerosis) cases show a family history, and the remaining 90% are sporadic. In 2018, through genome sequencing using two independent approaches, KIF5A was described as a novel ALS-associated gene. To describe clinical and genetic characteristics of a series of patients with motor neuron disease (MND), diagnosed at University Hospital of Palermo, carrying KIF5A variants. During 2019–2023, two hundred twenty-four patients with MND and healthy subjects with familial history of MND, underwent next-generation sequencing (NGS) for molecular analysis, including genetic testing for C9orf72 hexanucleotide-repeat expansion. The most mutated ALS genes, including KIF5A, were included in a NGS panel. Of the entire tested population, eight patients (including a brother and a sister) were found to carry KIF5A variants. Four patients had familial ALS, the other four were sporadic. Six patients were females (75%). Mean age at ALS onset was 59 years (33–75). Patients were evaluated according to the ALSFRS-revisited during follow-up visits. According to disease progression rate, five patients were defined as ∆FS ≤ 0.5 (slow-progressors), the remaining three patients showed a ∆FS > 1 (fast-progressors). Of the seven KIF5A variants, three are not already described in literature (respectively c.170 C > T, p.Thr57Met; c.2920T > G, p.Ser974Ala and c.2732 A > C, p.Lys911Thr). Two patients showed the association of variations in KIF5A with variations or mutations in other ALS genes, one of them carried a pathogenic variant of FUS (P525L). This study demonstrates phenotypic variability related to mutations in different regions of the same gene resulting in a susceptibility for the disease spectrum with different characteristics.

## Background

Amyotrophic lateral sclerosis (ALS), also known as motor neuron disease, is characterized by the degeneration of both upper and lower motor neurons, which leads to muscle weakness and death due to respiratory failure, typically within two to four years of symptom onset [1]. Approximately 10% of ALS show a family history (FALS) whereas the remaining 90% of ALS cases are sporadic (SALS). In 2018, Aude Nicolas et al. described KIF5A as an ALS-associated gene with genome-wide significance through two independent approaches.

Kinesin superfamily proteins (KIFs) and cytoplasmic dyneins are molecular motors that enable respectively anterograde and retrograde transport [2, 3]. Kinesin-1 is a heterotetramer composed of two light chains (KLC) and two heavy chains (KIF5) [4]. 

KIFs generally consist of a conserved motor domain, a dimerized coiled-coil domain, and a cargo-binding tail domain. The motor domain exhibits microtubule-stimulated ATPase activity, which allows the protein to move along microtubules [5]. Each KIF has a specialized tail domain [3] that binds to specific cargo vesicles and protein complexes [6]. KIF5A transport protein complexes and membrane organelles such as neurofilaments, RNA granules and mitochondria [6]. 

Several studies have demonstrated that KIF5A mutations are associated with hereditary spastic paraplegia (SPG), Charcot-Marie-Tooth neuropathy type 2 (CMT2) and ALS with different clinical phenotypes. Mutations contributing to SPG10 (spastic paraplegia 10) and to CMT2 are almost exclusively to be missense changes and are in the N-terminal motor domain (amino acids 9–327) of KIF5A. The mutations identified as contributing to ALS are found predominantly in the C-terminal cargo binding region of KIF5A (amino acids 907–1032) with the highly penetrant FALS mutations showing loss of function. Although the precise molecular mechanism is not known, previous studies have suggested that ALS- associated mutations in KIF5A are loss-of-function mutations because of the deletion of the cargo-binding tail domain (amino acids 907–1032) [7–9]. Nevertheless, the product of ALS-associated KIF5A variants KIF5A(Δexon27), resulting in exon 27 skipping, predisposed to form oligomers and aggregates [11]. KIF5A transport protein complexes and membrane organelles such as neurofilaments, RNA granules and mitochondria. Previous studies have suggested that ALS-associated mutations in KIF5A are loss-of-function mutations because of the deletion of the cargo-binding tail domain. The product of ALS-associated KIF5A alleles, KIF5A(Δexon27), is predisposed to form oligomers and aggregates [11]. Interestingly, KIF5A(Δexon27) oligomers showed higher motility than those of wild-type KIF5A regardless of the presence of KLC1 [10]. These data suggest that some ALS-associated mutations in KIF5A could be toxic gain-of-function mutations rather than simple loss-of-function mutations. It is noting that protein aggregates are often associated with neurodegenerative disorders [12] and KIF5A-(Δexon27) co-aggregates with wild-type KIF5A and KIF5B motors in the cell [10] while wild-type KIF5A has a propensity to form more aggregates in the cell than KIF5B. This propensity of KIF5A to oligomerize, similarly to the property that has been found for TDP-43, may be enhanced by ALS-associated KIF5A mutations and related to sporadic ALS [10]. In this study, we describe the clinical phenotype of patients carrying KIF5A variations from a series of patients collected from 2019 to 2023 and discuss their possible involvement in the pathogenesis of the disease.

## Methods

During the period 2019–2023, all consecutive ALS/MND patients referred to our Centre were included in the genetic study. Our study follows the principles of the Declaration of Helsinki. All patients have given written consent to the inclusion of material pertaining to themselves, they acknowledge that they cannot be identified via the paper, and we had fully anonymized them.

DNA extraction, from fresh blood samples, was performed at our neurochemistry laboratory with the use of a QIAGEN optimized DNA extraction kit, stored in a freezer at −20 °C and later sent through a temperature-controlled shipment appropriate for biological material.

Genetic analysis was performed in two different genetics laboratories using two different next-generation-sequencing (NGS) panels, respectively investigating 19 ALS-related genes and 145 ALS-related genes (the lists of genes are available on request) and including genetic testing for C9orf72 hexanucleotide repeat expansion.

During the period 2019–2023, two hundred twenty-four patients were screened for genes implicated in ALS etiology that included search for KIF5A. We found 8 patients with MND carrying KIF5a variations. (Table 1 and 2)

**Table 1:** Demographic characteristics of the studied cohort

Characteristics	N(%) or Mean
Total patients	224
Healthy subjects	21 (9.4%)
Gender	127 M (56.7%)
	97 W (43.3%)
Clinical diagnosis	Bulbar ALS 55 (24.6%)
	Spinal ALS 140 (62.5%)
	ALS-FTD: 2 (0.9%),
	PLS:6(2.7%)
Familial ALS	23 (10.3%)
Mean age at genetic screening/diagnosis	62 years
Geographical distribution	West Sicily: 106 M/81 W(total 187,83.5%)
	Center of Sicily: 10 M/5 W (15,6.7%)
	East Sicily: 7M/2 W (9, 4.0%)
	Other Italian regions/Other countries: 4 M/9 W (13, 5.8%)

*W *Women, *M* Men

**Table 2:**
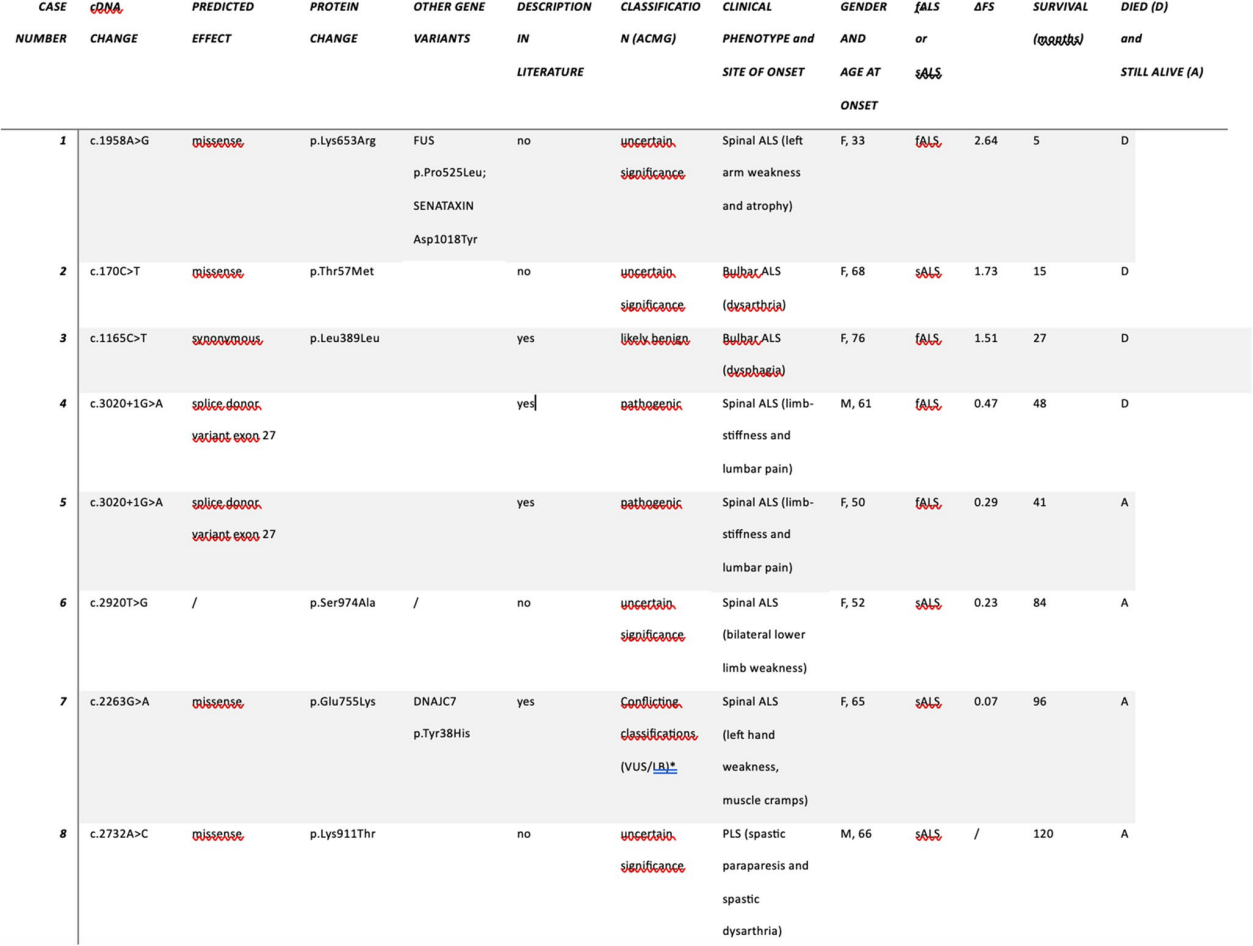
Description of case series and Kif5a variants

*W* Women,* M* Men

## Results: Description of the clinical series

### Case 1

Woman with familial Autosomal Dominant ALS linked to FUS P525L mutation (mother and two sisters died at a young age) and disease onset at age 33. Disease onset was in 2021, following a local adverse reaction to vaccine for Sars-Cov2 with cervicalgia and left brachialgia. Genetic testing documented, in addition to FUS P525L mutation, the presence of two VUS (variants of uncertain significance) in heterozygosity in SENATAXIN ASP1018Tyr (c.3052G > T) and KIF5A Lys653Arg (c.1958 A > G). It is not known whether the relatives also had these variants in association. Soon after, scapular girdle weakness began, left-side worse, with proximal atrophy. The disorder rapidly worsened to spastic paraparesis. Eventually, the patient died 5 months after disease onset from severe respiratory failure and hypercapnia. That was a very rapid form of disease with mean ΔFS = 2.64, calculated from the first visit (diagnosis) to follow-up assessments. This case is described based on the presence of a KIF5a gene-associated VUS, although it is known that the FUS P525L pathogenic mutation is closely linked to this juvenile-onset and rapidly progressive disease phenotype.

### Case 2

68-year-old woman developed mild hypophonia, flaccid dysarthria, and slight dysphagia for solids. She had an ENT (ear-nose-throat) examination, cervical and brain MRI which showed no abnormalities. She was referred to us for suspected MND and she was diagnosed with bulbar ALS. No family history of motor neurons disease. Over the months she worsened, swallowing became increasingly difficult to the point of needing a PEG implant. Her speech became severely dysarthric, but she did not report limb disorders. She presented marked emotional lability. She began to require assistance by continuous noninvasive ventilation. And finally, the possibility of tracheostomy was discussed, given the need for 24-hour ventilation. The patient had a form of ALS with rapid disease progression, mean ΔFS = 1.73. Genetic testing documented the presence of a KIF5a Thr57Met variant of uncertain significance. She died 15 months after disease onset.

### Case 3

A female patient with bulbar ALS onset at age of 76. History of hypertension and dyslipidemia. She had a familiarity for ALS (sister, suffering from spinal ALS who never had genetic testing) and major psychiatric disorders (father and 2 sisters). Despite being vaccinated, she contracted COVID-19. After recovery she remained asthenic, but it was thought to be a consequence of the infection. However, three months later she started complaining of sensation of “knot” in the throat, evolved over time into clear dysphagia and flaccid dysarthria. Then, hyposthenia of the neck also appeared, with dropped head and pain. In the same period, she has been experiencing shortness of breath in clinostatic position, and she was admitted to Neurology Unit and discharged, after investigations, with a diagnosis of ALS. Genetic testing positive for a VUS in KIF5A gene p.Leu389Leu c.1165 C > T. Over time, the swallowing and phonation disorder worsened, and she had significant weight loss (about 20 kg in a year). She underwent a PEG implant and practices exclusively enteral nutrition with a tube feed pump. She used NIV during the day. This case also shows rapid progression of the disease, ΔFS = 1.51.

### Case 4

Male, age of onset 61 years. Medical history was positive for familial ALS (mother and cousins had KIF5a gene mutation) and HCV-related cirrhosis. Onset with gait impairment and speech disorder (mixed dysarthria). Genetic testing confirmed genetic mutation c.3020 + 1G > A at donor site of exon 27 splicing of KIF5A gene. Over time, the complaints remained stable, so did the breathing. However, for months the patient has been experiencing increased stiffness in the right arm and right lower limb. He has no swallowing problems. Intermediate-slow progression of disease (Mean ΔFS = 0.47).

### Case 5

Sister of case number 4, disease onset at age 50. She only has history of asthma. She has a strong family history of ALS (mother, maternal uncles, an older brother). She has been suffering from low back pain and leg stiffness since 2021. An initial neurology and neurosurgery consult led to MRI (negative) and an EMG/ENG (electromyography). A lumbar puncture shows increased light and heavy neurofilaments. In addition, she also performed the genetic test which confirmed the same mutation as her brother. At the next visit, the patient reported worsening stiffness in her legs, the appearance of leg spasms upon waking, lower back pain and numbness in her left hand associated with muscle weakness. She began to have difficulty with fine hand movements, holding objects and speech slowed slightly. Gait has worsened, so she uses a walker at home and supports herself with someone in outdoor movements. The mean ΔFS = 0.29, indicative of a slow progression of the disease.

### Case 6

Case of a woman with an onset of MND at 52 years old. Patient is affected by a significant gonarthrosis. Neurological family history is characterized by Alzheimer’s disease, no family members with MND. For over a year she has been complaining of lower extremities strength deficit, first appearing on the right and then on the left side. The deficit was distal, with unsteady, stepping gait. She has fallen several times. She walks with single support (crutch). Following anesthesiologic examination for right knee replacement surgery, abnormalities worthy of neurologic evaluation were found. The patient underwent electromyography documenting bilateral distal denervation and she was referred for suspicion of motor neuron disease. She did not refer dysphagia or dysarthria, nor dyspnea. Genetic test found KIF5A mutation p.Ser974Ala. Mean ΔFS = 0.23, indicative of slowly progressing disease.

### Case 7

A 69-years-old woman with subtle onset of hyposthenia and atrophy of the left hand that appeared about 4 years earlier, at the age of 65. This condition progressively worsened until it resulted in difficulty buttoning, turning a key, and picking up small objects. The clinical picture at diagnosis showed left hand and forearm “en-masse” atrophy, with claw hand, fasciculations, and hypereflexia also in the atrophic limb. No neurological signs were present in the other limbs or in the bulbar district. The patient reported a medical history characterized only by migraine in youth, which regressed after menopause, and muscle cramps as early as a few years before disease onset extended to the upper limbs and abdomen. Family history was positive for neurological (multiple sclerosis) and oncological diseases. The patient underwent clinical checkups and instrumental examinations over the years, a cervical MRI documented spondylosis with unremarkable protrusions and hernias and electro-neurophysiological examinations showing neurogenic distress and reduced cMAP amplitude in ulnar and median nerves. A diagnosis of MND was made. After genetic testing, two variations in heterozygosity were found, respectively in the KIF5A p.Glu755Lys gene (c.2263G > A) and in the DNAJC7 p.Tyr38His gene (c.112T > C). The patient, over the years, manifested a very slow progression of disease, with an average ALSFRS of 42/48 and ΔFS = 0.07, she never manifested cognitive-behavioral impairments.

This case is reported as exploratory since a conflicting classification (uncertain significance/likely benign) KIF5a variant was incidentally detected.

### Case 8

A male patient with an onset at the age of 66 years old. For about 9 years, he had spastic dysarthria and gait disturbances. One year before the onset of disease he was involved in a traffic accident causing a pelvic fracture. Followed by other neurological specialists over the years, he was investigated for different neurodegenerative disorders (spinocerebellar ataxia, spastic paraparesis, amyotrophic lateral sclerosis). Finally, clinical presentation appeared compatible with slow-evolving motor neuron disease (suspected PLS or HSP) for the presence predominantly of first motor neuron signs. Over the years, he reported worsening of motor, balance, and swallowing disorders, and a change in behaviour. The result of the genetic test showed the presence of a variant in heterozygosity of the KIF5A gene p.Lys911Thr (c.2732 A > C).

## Discussion

We describe 8 patients carriers of KIF5a variants/mutation affected with different phenotypes of ALS and motor neurons disease. These patients represent 3.6% of the entire screened cohort but considering the presence in the case series of two siblings carrying the same mutation and the case of a patient with conflicting classification variant (VUS/LB), this percentage drops to 2.7% (1.6% in SALS population and 13% in FALS population). Four of the eight patients had familial ALS (two were siblings), the other four were sporadic. Six patients were females (75%). Mean age at ALS onset was 59 years (according to the mean age at onset of all population). As expected, diagnostic delay (DD) varied based on symptoms onset. Five patients had a spinal onset with a DD of 23 months, two had a bulbar onset with a 7 months DD and only one patient had a primary lateral sclerosis (PLS) with DD of 60 months.

At the time we describe this series, 3 patients are dead (mean disease duration was 22.6 months, 5–48). Of the five patients who are still alive, mean follow-up is 74 months (27–120). Patients were evaluated according to the ALSFRS-revisited during every follow-up visit. At baseline (first visit), regarding disease duration at the time of assessment, five patients were defined as slow-progressors with ∆FS ≤ 0.5, while the remaining three patients showed a ∆FS > 1 (fast-progressors). The ∆FS expressed in the case descriptions derived from an average based on the first visit assessment (at the time of diagnosis) and further clinical follow-ups.

Some studies have described the clinical phenotype of patients with KIF5A mutations/variants. Eva M. J. de Boer described a case of a 38-year-old man presented with a four-year history of progressive weakness of the lower limbs and inability to lift his left leg. Thanks to a genetic screening was found an heterozygous variant c.1702G > A p.(Gly568Arg) in the KIF5A gene. The proband’s father had similar symptoms with weakness and stiffness of the lower limbs and it was revealed that he was also heterozygous carriers of the same variant [13]. 

Nakamura et al., in 2020, described 13 patients harbored a nonsynonymous variant, and one patient harbored a splice-site variant in the KIF5A gene. In that study, 4 patients carried the same heterozygous missense variant (p.Asp853Asn), 2 patients carried another heterozygous missense variant (p.Arg716Gln), and 7 patients carried different heterozygous missense variants (p.Pro46Ala, p.Arg423His, p.Leu494Met, p.Thr585Ala, p.Arg588Gln, p.Glu881Lys, and p.Glu1028Asp). The patient with the novel splice-site variant (c.2993–3 C > A) in the KIF5A gene, presented an onset at the age of 55 with dropped head, and progressive upper limbs weakness [14]. 

Another study described two mutations in KIF5A (p.Glu251Lys and p.Gly235Glu) that have been identified in patients with an isolated axonal neuropathy but no pyramidal signs, compatible with a clinical diagnosis of CMT2 and with hereditary spastic paraplegia. These findings have raised the hypothesis that KIF5A mutations have a broad phenotypic spectrum and that SPG10 and CMT2 may be allelic disorders. Variable phenotypes in individuals within the same family were also observed. A patient with D232N mutation, presented a complex CMT2 associated with spasticity, whereas his brother had pure HSP. Classical or complex CMT2 is relatively rare, while HSP with axonal neuropathy is the commonest phenotype associated with KIF5A mutations. However, a specific phenotype–genotype correlation for KIF5A mutations could not be defined as some mutations have been associated with more than one phenotype [15]. 

Finally, Crimella C et al., performed a genetic screening of KIF5A gene in a series of 139 HSP and 36 CMT2 affected subjects to confirm its involvement in both CMT2 and SPG10 phenotypes. They identified five missense changes, four in five HSP patients and one in a CMT2 patient. All mutations were localized in the kinesin motor domain except for one in the stalk domain and predicted to generate protein structure destabilization [16]. 

The study of this series of patients allows us to highlight how the presence of KIF5A gene polymorphisms is often found in the ALS population, regardless of clinical phenotype and age of onset. Nevertheless, except for patients 3 and 4, who carry a gene variant already known to be pathogenic, we lack biological evidence to identify the reported variants as causative mutations. However, this focus on KIF5A calls to mind all the VUS that are often found in MND-related genes, whose actual molecular impact and clinical burden are still unknown. For this reason, we believe that the description of such findings is important to better understand not only the possible role in disease pathogenesis but the impact they may have on progression, age of onset, and survival. The genetic variants reported by our patients do not show the typical distribution associated with spastic paraplegia and CMT in the N-terminal region of the protein compared to C-terminal involvement typical of MND forms. In fact, even patients with polymorphisms present in the N-terminal region manifested a more aggressive phenotype of motor neuron disease; conversely, one patient (#8), despite having a genetic variant in the C-terminal region of the gene manifested for years a phenotype compatible with an MND variant of disease that involved exclusively the first motor neuron and with a slow evolution (primary lateral sclerosis), similar to what is expected in a clinical phenotype of HSP (which he was in fact mis-diagnosed with). Additionally, it may be important to investigate whether the presence of additional genetic variants in patients modulates the clinical presentation.

This study illustrates phenotypic variability related to variants in different regions of the same gene resulting in a susceptibility for the disease spectrum with different characteristics. Our findings expand the current understanding of this gene in the ALS population, confirming its marked phenotypic heterogeneity and the lack of a consistent genotype–phenotype correlation. Notably, cases of siblings carrying the same pathogenic mutation but presenting distinct clinical manifestations further support this observation. This variability suggests that additional genetic, epigenetic, and environmental factors may modulate disease onset and progression.

## Limitations of the study

Our study has some limitations: the relatively small study population and its lack of heterogeneity because originating from a small region of Italy, such as Sicily, and the absence of functional molecular biology studies to understand the possible pathogenicity of the VUS detected.

Neuropsychological and cognitive tests were unfortunately not available for all patients described, as some deceased prior to manuscript preparation, precluding collection data. For this reason, we have chosen to not include this aspect in the description of the clinical cases.

Nevertheless, the description of clinical cases in the case of an uncommon/rare disorder still plays an essential role in scientific awareness of the disease and its associated clinical phenotypes. It allows us to understand their correlation or manifestation in the presence of a particular genetic variant. It still allows us to share our observations with the scientific community with the aim of having in the future the possibility of demonstrating a true cause-and-effect association and a possible genotype-phenotype link. This would also increase our knowledge of such a complex and still poorly understood pathology as ALS and motor neuron diseases and allow us to better manage it.

In our series we also observed that KIF5a variants are present at a frequency of 2.7% (in a population composed by both familial and sporadic ALS), which is higher than reported in gnomAD for healthy non-Finnish Europeans [17] and in other studies conducted in Chinese populations [18] and similar to the frequency found in European cohorts and in Japanese studies in which almost 1.7% of the SALS patient were carrier of a KIF5a variant [14, 19]. This suggests a higher occurrence of genetic variants of KIF5a in European patients, even as rare as in the other countries.

## Data Availability

The data that support the findings of this study are available on request from the corresponding author. The clinical data are not publicly available to protect information that could compromise the privacy of research participants.
